# Improving the measurement of semantic similarity by combining gene ontology and co-functional network: a random walk based approach

**DOI:** 10.1186/s12918-018-0539-0

**Published:** 2018-03-19

**Authors:** Jiajie Peng, Xuanshuo Zhang, Weiwei Hui, Junya Lu, Qianqian Li, Shuhui Liu, Xuequn Shang

**Affiliations:** 1 School of Computer Science, Northwestern Polytechnical University, Xi’an, China; 2 Key Laboratory of Big Data Storage and Management, Northwestern Polytechnical University, Ministry of Industry and Information Technology, Xi’an, China; 3 Centre for Multidisciplinary Convergence Computing (CMCC), School of Computer Science, Northwestern Polytechnical University, Xi’an, China

**Keywords:** Gene Ontology, Semantic similarity, Random walk with restart

## Abstract

**Background:**

Gene Ontology (GO) is one of the most popular bioinformatics resources. In the past decade, Gene Ontology-based gene semantic similarity has been effectively used to model gene-to-gene interactions in multiple research areas. However, most existing semantic similarity approaches rely only on GO annotations and structure, or incorporate only local interactions in the co-functional network. This may lead to inaccurate GO-based similarity resulting from the incomplete GO topology structure and gene annotations.

**Results:**

We present NETSIM2, a new network-based method that allows researchers to measure GO-based gene functional similarities by considering the global structure of the co-functional network with a random walk with restart (RWR)-based method, and by selecting the significant term pairs to decrease the noise information. Based on the EC number (Enzyme Commission)-based groups of yeast and Arabidopsis, evaluation test shows that NETSIM2 can enhance the accuracy of Gene Ontology-based gene functional similarity.

**Conclusions:**

Using NETSIM2 as an example, we found that the accuracy of semantic similarities can be significantly improved after effectively incorporating the global gene-to-gene interactions in the co-functional network, especially on the species that gene annotations in GO are far from complete.

## Background

Recently, significant improvement in high-throughput biology technologies has led to an exponential increase in biological data. Gene Ontology (GO) is one of the most popular bioinformatics resources used to interpret the result of biological experiment. GO provides structured, controlled vocabulary of terms to describe genes by three types of attributes that are molecular function, biological process and cellular component [1]. In each category, terms are structured as a directed acyclic graph (DAG). GO provides a convenient and important way to study functional similarity. GO-based semantic similarity has been successfully used in many research areas, such as gene function prediction [2–5], gene network analysis [6, 7], homology analysis [8], gene association visualization [9] and missing value imputation [10, 11].

In the past decade, a lot of approaches have been proposed to calculate gene functional similarity based on gene ontology [12–23]. Based on the information used in similarity calculation, these measurements can be loosely classified into four groups: path length-based methods, node-based methods, integrative methods and network-based methods.

The methods in the edge-based group calculate similarity by considering the topology structure information of GO [24, 25]. A recently proposed approach, named Relative Specificity Similarity (RSS), takes two types of length information into account: the edge length from given term pair to their closest leaf terms; and the edge length to their lowest common ancestor (LCA) [25]. The experiment result shows that this method is superior in correlation with sequence and Pfam similarities. However, the edge-based methods are fully relied on the topology of GO DAG. This type of methods cannot differentiate the terms at the same topological level [14].

For the node-based methods, the approaches rely on the specific taxonomy. One of the proposed approaches exploit the information content (IC) of the most informative common ancestor (MICA) to measure the similarity between two GO terms [26]. Let *t* be a MICA term. We calculated its IC as −*log*(|*G*_*t*_|/|*G*_*root*_|). *G*_*t*_ and *G*_*root*_ represent gene sets annotated to term *t* and *root* respectively. This method is further improved by taking the path length from the term pair to its MICA into account [12]. The evaluation test shows that the results are consistent with protein sequence similarities. However, node-based approaches only take the annotations into account, ignoring the topology information of the GO.

In the integrative group, the approaches are proposed to use more information in GO. Hybrid Relative Specificity Similarity (HRSS) uses four types of information (information content, structure topology, annotations and MICA) to calculate the semantic similarity [25]. InteGO method proposed a rank-based method to integrate multiple existing similarity methods, called seed methods, to consider more aspects of GO [17]. InteGO2 method selects the most appropriate methods from a set of methods by a voting method and integrates these selected methods based on a metaheuristic search method [9]. The evaluation test shows that the integrative method performs better than the seed method. However, all these methods are only based on the GO, neglecting the inaccurate representation and missing information of GO. For example, 37% of the Arabidopsis genes have experimental annotations of all three domains of GO [27]. Therefore, low-quality similarity may result from the incomplete information in GO.

A network-based method, called NETSIM, was recently proposed to address these problems by integrating gene-gene associations and GO topology structure and annotations [19]. The experiment based on metabolic reaction map shows that semantic similarity can be enhanced by incorporating gene-gene associations. Unfortunately, only part of the information in gene co-function network was used, since NETSIM only considered the direct link in the network. Other than the directly connected gene pairs, the indirect gene-gene interactions contained in the gene co-function network should also be considered. However, considering indirect interactions may also import the noise information.

In this paper, we proposed a novel network-based method named NETSIM2, by considering both direct and indirect interactions in the gene co-function network with a random walk based method, and by selecting the significant term pairs for similarity calculation to decrease the effect of the imported noise information. Comparing with the existing approaches, NETSIM2 has the following advantages:

- Comparing with the state-of-art methods, NETSIM2 performs better than existing methods by incorporating gene co-functional network effectively.
- A random walk with restart-based method is developed to take both direct and indirect interactions into account.
- A standard score-based method is proposed to select the significant GO-term pairs to measure the semantic similarity.

## Methods

**Fig. 1:**
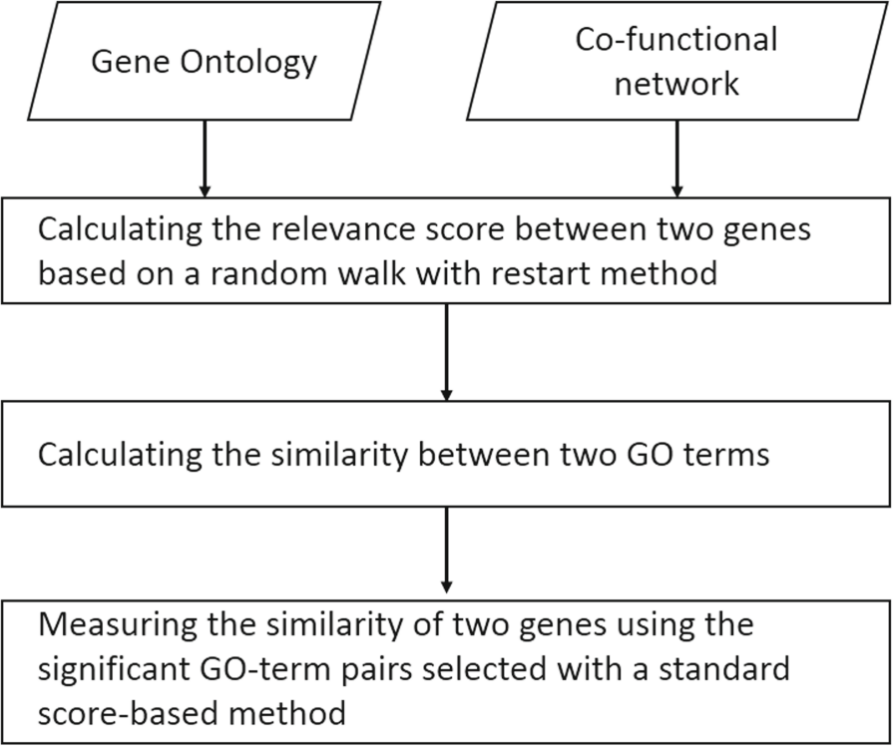
The workflow of *NETSIM*2

NETSIM2 calculates the semantic similarity between two genes in three steps (see Fig. 1). First, given a gene co-functional network, it computes the relevance score between two genes based on a random walk with restart method. Second, it calculates the similarity between two GO terms by combining the information from co-functional network and GO. Finally, it selects the significant GO-term pairs to measure the similarity of two genes using a standard score-based method.

### Calculating the relevance score between genes

In this step, we consider both the direct and indirect interactions in the gene co-functional network to calculate the relevance score between two genes. A gene network includes not only the direct interactions but also the associations between indirectly connected genes. In this step, we adopted the random walk with restart (RWR) [28] algorithm to measure the relevance score between two genes. The relevance score between genes could be represented by the stationary probability calculated by RWR. Comparing with the direct interactions, the relevance score defined by RWR can capture the global structure information of the co-functional network [29]. Furthermore, comparing with the graph distance metrics (such as shortest path), it can reveal the multi-facet relationship between two genes [30].

In RWR method, a random process begins from gene *i*. It iteratively transmits to neighbors of *i* with the probability that is based on the weights of edges. Similarly, the particle has the probability *c* to go back to start gene *i*. The association score between gene *i* and gene *j* could be defined as the stationary probability *r*[*i*,*j*] that the iteration process will finally stop at gene *j*. Mathematically, given a co-functional network *N*(*V*,*E*), the relevance scores between genes can be calculated by following steps. First, given a weight matrix *M* corresponding to *N*, a normalized weighted matrix *M*^′^ was generated. Then, the RWR-based method could be described as follows.

1 $$ \mathbf{r}_{i+1} = cM^{\prime}\mathbf{r}_{i} + (1-c)\mathbf{e}_{i}  $$

where **r**_*i*_ is a |*V*|×1 vector and **e**_*i*_ is a |*V*|×1 starting vector (the *i*^*th*^ element is 1 and others 0). (1−*c*) is defined as the restart probability, which is between 0 and 1. Based on Equation 1, **r**_*i*_ can be defined as follows.

2 $$ \mathbf{r}_{i} = (1-c)(\boldsymbol{I} - cM^{\prime})^{-1}\mathbf{e}_{i}  $$

After this step, we can get a matrix *R*, which saved the relevance scores between each pair of genes in *N*(*V*,*E*).

### Calculating the similarity between two GO terms

In this step, we calculate the similarity between two GO terms combining the information from co-function network and GO based on the method we represented in our previous work [19].

Let *t*_1_ and *t*_2_ be two terms. We define *D*(*t*_1_,*t*_2_) as the gene set distance to compute the similarity between sets of genes annotated by *t*_1_ and *t*_2_. *D*(*t*_1_,*t*_2_) is defined as:

3 $$ {\begin{aligned} D(t_{1},t_{2}) = \frac{\sum_{g_{i} \in G_{1}}\prod_{g_{j} \in G_{2}}d_{ij}+\sum_{g_{i} \in G_{2}}\prod_{g_{j} \in G_{1}}d_{ij}}{2|G_{1} \cup G_{2}| - \sum_{g_{i} \in G_{1}}\prod_{g_{j} \in G_{2}}d_{ij}-\sum_{g_{i} \in G_{2}}\prod_{g_{j} \in G_{1}}d_{ij}} \end{aligned}}  $$

where *G*_1_ and *G*_2_ are the gene sets annotated by *t*_1_ and *t*_2_ respectively. *d*_*ij*_ is the distance score between two genes, *d*_*ij*_=1−*R*_*ij*_. *R*_*ij*_ is the relevance score between gene *i* and *j* calculated by RWR-based method. The gene set distances of all term pairs are normalized between 0 and 1.

Then, we calculate the similarity between two terms based on a “path-constrained annotation”, labeled as *U*. In traditional lowest common ancestor (LCA)-based methods, all the descendants of LCA are considered. The “path-constrained annotation" method only uses the terms that are the most relevant to the compared terms. The set of relevant terms includes three parts: the gene set annotated by term *t*_1_ and *t*_2_, and the gene set annotated by the common parent *p* of *t*_1_ and *t*_2_ and its descendant terms that are on the paths from *t*_1_ or *t*_2_ to *p*.

Let *t*_1_ and *t*_2_ be two GO terms and *p* be their common ancestor. Then, the similarity between *t*_1_ and *t*_2_ is defined based on the equation proposed in our previous work [19].

4 $$ \begin{aligned} S(t_{1},t_{2})&=\frac{2log|G|-2logf(t_{1},t_{2},p)}{2log|G|-(log|G_{1}|+log|G_{2}|)}\\ &\quad \times\left (1-\frac{h(t_{1},t_{2})}{|G|}\times \frac{G_{p}}{G}\right) \end{aligned}  $$

where *G*_*p*_ (or G) is the gene set annotated by common ancestor term *p* (or root term) and its descendants. In the equation, *f*(*t*_1_,*t*_2_,*p*) calculates the similarity based on the path-constrained annotations, and is defined as follows.

5 $$ {{} \begin{aligned} f(t_{1},t_{2},p) &= D(t_{1},t_{2})^{2} \times |U(t_{1},t_{2},p)| + \left(1-D(t_{1},t_{2})^{2}\right) \\ &\quad\times \sqrt{|G_{1}| \times |G_{2}|} \end{aligned}}  $$

*h*(*t*_1_,*t*_2_) measures the specificity of the common parent, and is defined as follows.

6 $$ \begin{aligned} h(t_{1},t_{2}) = D(t_{1},t_{2})^{2} &\times |G| + \left(1-D(t_{1},t_{2})^{2}\right) \\ &\quad \times max(|G_{1}|,|G_{2}|) \end{aligned}  $$

In Eq. 4, the left part measures the distance from term *t*_1_ and *t*_2_ to *p*, and the right part calculates the distance from *p* to root. It is noted that we selected the highest score as the similarity between *t*_1_ and *t*_2_, if there are more than one lowest common ancestor.

### Measuring the similarity of two genes

Considering both the direct and indirect interactions in the gene co-functional network may import noise information. In this step, to decrease the noise, we select the significant term pairs to calculate the gene similarities.

Let *g*_*i*_ and *g*_*j*_ be two genes. *T*_*i*_ and *T*_*j*_ are the annotation sets of *g*_*i*_ and *g*_*j*_. Let *T*_*G*_ be the set of all terms contained in a GO category. Given a term *t*, we calculate similarities between *t* and each term in *T*_*G*_/*t*, saved as *S*_*t*_. Let *t*^′^ be a term in *T*_*G*_/*t*. The standard score of similarity $z_{t,t^{\prime }}$ is defined as follows.

7 $$ z_{t,t^{\prime}} = \frac{S(t,t^{\prime}) - \mu_{t}}{\sigma_{t}}  $$

where *μ*_*t*_ is the mean of the *S*_*t*_ and *σ*_*t*_ is the standard deviation of *S*_*t*_. If |*z*_(_*t*,*t*^′^)| is larger than 1.6 (*p*−*value* is less than 0.05), pair (*t*,*t*^′^) is considered as a significant term pair.

The gene similarity are calculated as follows:

8 $$ {{} \begin{aligned} GeneSim(g_{i},g_{j}) = \frac{\sum_{t \in T_{i}}Sim\left(t,T_{j}^{\prime}\right) + \sum_{t \in T_{j}}Sim\left(t,T_{i}^{\prime}\right)}{|T_{i}| + |T_{j}|} \end{aligned}}  $$

where $T_{j}^{\prime }$ ($T_{i}^{\prime }$) is the term set selected from *T*_*j*_ (*T*_*i*_). To test the similarity between term *t*∈*T*_*i*_ and term set *T*_*j*_, we first select a term set $T_{j}^{\prime }$ from *T*_*j*_. Based on the standard score, given term *t*, we can select two significant sets from *T*_*j*_: $T_{th}^{\prime } =\left \{t^{\prime }| \left (z_{t,t^{\prime }} > 1.6\right)\right \}$ or $T_{tl}^{\prime } =\left \{t^{\prime }| \left (z_{t,t^{\prime }} < -1.6\right)\right \}$. If $\left |T_{th}^{\prime }\right | > \left |T_{tl}^{\prime }\right |$, then $T_{j}^{\prime } = T_{th}^{\prime }$, else $T_{j}^{\prime } = T_{tl}^{\prime }$. $T_{i}^{\prime }$ is obtained in the similar way. Choosing the significant terms to calculate the gene similarity can decrease the noise information. Each term *t*∈*T*_*i*_(*T*_*j*_) can find at least a term in *T*_*j*_(*T*_*i*_) to make a significant term pair. For each *t*∈*T*_*x*_, $Sim\left (t,T_{y}^{\prime }\right) ={max}_{t_{y} \in T_{y}^{\prime }}S\left (t,t_{y}\right) $.

## Results and discussion

### Data preparation

We downloaded the GO structure and annotations from GO website in Dec. 2016 (www.geneontology.org). In our work, only the is-a and part-of relationships were used. We used gene associations included in YeastNet [31]and AraNet [32] for evaluation test on yeast and arabidopsis respectively. The EC group of Yeast and Arabidopsis were downloaded from http://www.yeastgenome.org/ and http://ftp.plantcyc.org/Pathways respectively.

### Performance evaluation criteria

NETSIM2 is evaluated based on the EC number (Enzyme Commission) group information, which has been used in previous research [18]. The idea is that genes that are labeled by the same EC number have the similar function. Genes are grouped to different categories based on their EC numbers (full four digits). Then, we test whether the genes in the same category have higher similarity than genes in different categories. Mathematically, we use the logged fold change (LFC) measure [18] for quantitative evaluation. The LFC score of EC number *e*_*i*_ is calculated as follows:

9 $$  LFC(e_{i})= \frac{1}{|EC|} \times \sum\limits_{e_{j} \in EC; G(e_{j})\cap G(e_{i}) = \emptyset}{\frac{\sum\limits_{g \in G({e_{i}})} {diff}_{g}(e_{i},e_{j})}{|G({e_{i}})|} }  $$

where *G*(*e*_*i*_) is gene set that includes genes labeled by *e*_*i*_; *EC* is a set of ECs satisfying that no annotated genes is included in *e*_*i*_ (*G*(*e*_*j*_)∩*G*(*e*_*i*_)=*∅*); and *diff*_*g*_(*e*_*i*_,*e*_*j*_) is defined as:

10 $$ {\begin{aligned} {diff}_{g}(e_{i},e_{j})=\ln{\frac{ |G(e_{i})| \times \sum\limits_{g^{\prime} \in G(e_{j})}{\left(1-GeneSim(g,g^{\prime})+c\right)}}{ |G(e_{j})| \times \sum\limits_{g^{\ast} \in G(e_{i})}{\left(1-GeneSim(g,g^{\ast})+c\right)}}} \end{aligned}}  $$

*G*(*e*_*i*_) is the gene set of *e*_*i*_ without *g*; *G*(*e*_*j*_) is the gene set of *e*_*j*_; where *c* is a Laplacian smoothing parameter; *g* is a gene assigned to *e*_*i*_. *GeneSim*(*g,g*^′^) and *GeneSim*(*g,g*^∗^) are defined in Eq. 8. Equation 10 measures the difference between the inter-EC distance and intra-EC distance.

### Performance evaluation on molecular function category

The performance of *NETSIM*2 was evaluated by comparing the GO-based similarity between genes in different EC categories and same category. In this subsection, the gene similarities are calculated based on molecular function category and co-functional network. We used LFC score as a criteria to compare five measures (Resnik [33], Relevance [12], Wang [13], NETSIM [19] and *NETSIM*2) on both yeast and arabidopsis data.

**Fig. 2:**
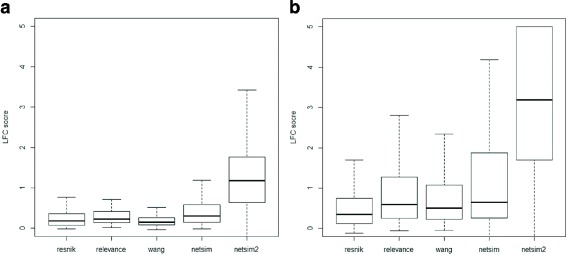
Performance comparison of different measures on GO’s molecular function terms in yeast (**a**) and Arabidopsis (**b**)

**Fig. 3:**
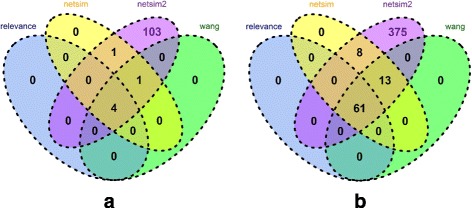
Number of ECs for which NETSIM2, NETSIM, Wang and Relevance measures performed the best for yeast (**a**) and Arabidopsis (**b**) based on molecular function terms

NETSIM2 performed the best in all tests. In yeast, the LFC score of NETSIM2 was the highest in all tested measures (Fig. 2a, Table 1). Specifically, the median, 75th and 25th percentile value of LFC scores of NETSIM2 on yeast were 1.18, 1.76 and 0.64, significantly higher than the other measures. Interestingly, the performance of NETSIM2 was significantly higher than our previous measure NETSIM, indicating that considering the global structure of co-functional network can improve the performance. Comparing the LFC scores on each EC group using NETSIM2, NETSIM, Relevance and Wang measure (top four measures), the result shows that NETSIM2 has the highest LFC score in all 109 ECs, while NETSIM, Relevance and Wang measure has the highest LFC score in 6, 4 and 5 ECs only (Fig. 3a).

**Table 1:** The LFC scores of five methods for the molecular function category on yeast data

Method	Resnik	Relevance	Wang	NETSIM	NETSIM2
25%	0.07	0.14	0.08	0.15	0.64
50%	0.18	0.23	0.15	0.31	1.18
75%	0.36	0.42	0.25	0.58	1.76

Similarly, the LFC score of NETSIM2 was the highest in all evaluated measures in arabidopsis data (Fig. 2b, Table 2). Figure 2b shows that NETSIM2 performed significantly better than other measurements in arabidopsis data. Specifically, the 75th percentile of NETSIM2 is 5, which is the highest in all tested methods. The score of NETSIM, Relevance, Wang and Resnik measure are 1.87, 1.27, 1.07 and 0.75 respectively. The 50th percentile of NETSIM2 is 3.19, which is about 5 times of the second best measure NETSIM (0.65). Comparing the LFC scores on each EC group using NETSIM2, NETSIM, Relevance and Wang measure (top four measures), the result shows that NETSIM2 got the highest LFC score in all 457 ECs, while the number for NETSIM, Relevance and Wang measure were 82, 61 and 74 respectively (Fig. 3b). It is noted that we set the higher bound of the LFC scores as 5.

**Table 2:** The LFC scores of five methods for the molecular function category on Arabidopsis data

Method	Resnik	Relevance	Wang	NETSIM	NETSIM2
25%	0.12	0.25	0.22	0.26	1.69
50%	0.35	0.59	0.51	0.65	3.19
75%	0.75	1.27	1.07	1.87	5

All these results indicate that NETSIM2 can improve the precision of semantic similarity measurement on molecular function category by incorporating co-function network effectively.

### Performance evaluation on biological process category

In this subsection, we evaluated NETSIM2 on the biological process category. The same LFC score (Eq. 9) were used in the performance evaluation. We also evaluated NETSIM2 on both yeast and arabidopsis data.

**Fig. 4:**
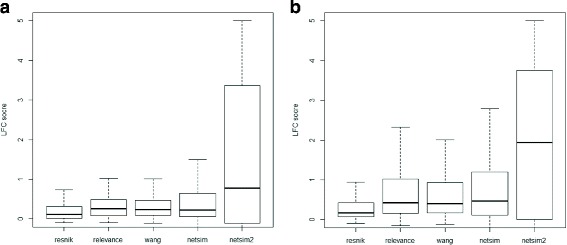
Performance comparison on LFC scores of similarity measures on GO’s biological process in yeast (**a**) and Arabidopsis (**b**)

**Fig. 5:**
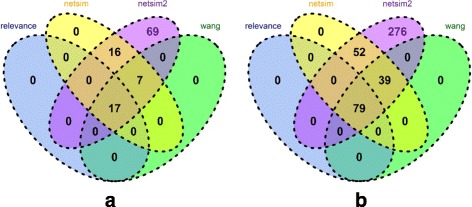
Number of ECs for which NETSIM2, NETSIM, Wang and Relevance measures performed the best for yeast (**a**) and Arabidopsis (**b**) based on biological process terms

Overall, NETSIM2 performed better than other four measures (NETSIM, Wang, Relevance and Resnik). In yeast, the 75th and median percentile of LFC scores were significant higher than other measures (Fig. 4a, Table 3), indicating that considering the global structure of co-function network and noise decrease can improve the overall performance. Specifically, the 75th percentile of LFC scores is 3.37, while the values of other measures are all less than 1 (0.64, 0.47, 0.49 and 0.31 for NETSIM, Wang, Relevance and Resnik respectively). Comparing the LFC scores on each EC group using NETSIM2, NETSIM, Relevance and Wang measure (top four measures), the result shows that NETSIM2 has the highest LFC score in all 109 ECs, while NETSIM, Relevance and Wang measure have the highest LFC score in 40, 17 and 24 ECs respectively (Fig. 5a).

**Table 3:** The LFC scores of five methods for the biological process category on yeast data

Method	Resnik	Relevance	Wang	NETSIM	NETSIM2
25%	0.01	0.08	0.10	0.06	0.11
50%	0.12	0.26	0.24	0.23	0.78
75%	0.31	0.49	0.47	0.64	3.37

Similarly, NETSIM2 performs the best in all tested measures based on biological process category in arabidopsis data (Fig. 4b, Table 4). The median and 75th percentile of LFC scores for NETSIM2 are 1.94 and 3.75, which are significant higher than the second-best measure NETSIM, which are 0.47 and 1.19 respectively (Fig. 4b and Table 4). In addition, Only NETSIM2 performs best in 276 ECs in the testing set arabidopsis ECs (Fig. 5b). For all ECs, NETSIM2 performs best, while the second best method performs best on 170 ECs.

**Table 4:** The LFC scores of five methods for the biological process category on Arabidopsis data

Method	Resnik	Relevance	Wang	NETSIM	NETSIM2
25%	0.07	0.15	0.17	0.12	0.002
50%	0.17	0.43	0.40	0.47	1.94
75%	0.42	1.03	0.91	1.19	3.75

In evaluation on both molecular function and biological process category, NETSIM2 improves more on arabidopsis data than yeast data. The reason may be that yeast data in GO is more complete than arabidopsis data. Therefore, incorporating co-functional network can improve the performance significantly on the arabidopsis data.

## Conclusions

Gene Ontology (GO) is one of the most popular bioinformatics resources used to describe the properties of genes and gene products. Calculating GO-based gene functional similarity has been widely used in multiple research areas. However, the low-quality similarity may result from the incomplete information of GO and the limited amount of annotations in GO. A recent measure, named NETSIM, addresses these problems by considering both gene-gene associations, GO DAG and annotations. Unfortunately, only the local association information in gene co-function network was used, since NETSIM only considers the direct link in the network.

In this paper, we proposed a novel network-based method, named NETSIM2, by considering the global structure of the co-functional network with a RWR-based method, and by selecting the significant term pairs to decrease the noise information. NETSIM2 includes three steps: firstly, given a gene co-functional network, the relevance scores between two genes are calculated based on a random walk with restart method; secondly, the similarity between two GO terms is calculated by combining the information from co-functional network and GO; finally, the significant GO-term pairs are selected to measure the similarity of two genes using a standard score-based method. Experimental results using ECs on both molecular function and biological process category show that NETSIM2 performs the best among all the measures on both yeast and Arabidopsis data set. It also shows that NETSIM2 can significantly improve the performance of semantic similarity measurement especially on the incomplete species. It is note that we have proposed NETSIM in our previous work to incorporate co-function network to GO-based semantic similarities, which can be considered as a simplified case of NETSIM2.
